# Receptors of Advanced Glycation End Product (RAGE) Suppression Associated With a Preserved Osteogenic Differentiation in Patients With Prediabetes

**DOI:** 10.3389/fendo.2022.799872

**Published:** 2022-02-14

**Authors:** Mattabhorn Phimphilai, Peraphan Pothacharoen, Nipon Chattipakorn, Prachya Kongtawelert

**Affiliations:** ^1^ Division of Endocrinology, Department of Internal Medicine, Faculty of Medicine, Chiang Mai University, Chiang Mai, Thailand; ^2^ Thailand Excellence Center for Tissue Engineering and Stem Cells, Department of Biochemistry, Faculty of Medicine, Chiang Mai University, Chiang Mai, Thailand; ^3^ Cardiac Electrophysiology Research and Training Center, Faculty of Medicine, Chiang Mai University, Chiang Mai, Thailand; ^4^ Department of Physiology, Faculty of Medicine, Chiang Mai University, Chiang Mai, Thailand

**Keywords:** advanced glycation end products, impaired fasting glucose, osteogenic differentiation, prediabetes, peripheral blood mononuclear cell, receptor of advanced glycation end products, type 2 diabetes

## Abstract

Type 2 diabetes is widely documented for osteogenic differentiation defect and impaired bone quality, which is related to the skeletal accumulation of advanced glycation end products (AGEs). Prediabetes is a condition in which hyperglycemia is lower than the threshold for the diagnosis of diabetes. Prediabetic animal models consistently demonstrate impaired osteogenic differentiation and deteriorated bone microarchitecture. However, no evidence shows defects in osteoblast development and skeletal effects of AGEs in prediabetic individuals. Therefore, it remains to be elucidated whether impaired osteogenic differentiation ability and altered cellular response to AGEs occur in patients with prediabetes. This cross-sectional study included 28 patients with prediabetes as defined by impaired fasting glucose criteria, fasting plasma glucose (FPG) between 100–125 mg/dl and 17 age-matched normoglycemic controls to elucidate osteogenic differentiation and *AGER* expression in the PBMC derived from those individuals. The PBMC-isolated from both groups showed similar rates of expression of osteoblast-specific genes, namely, *ALPL*, *BGLAP*, *COL1A1*, and *RUNX2/PPAR* (89.3% and 88.2%, *p* = 1.000), and showed comparable levels of expression of those genes. By using age- and pentosidine-matched normoglycemic individuals as references, the PBMC-isolated from prediabetic patients demonstrated lower expression of both *AGER* and *BAX/BCL2*. The expression of *AGER* and *BAX/BCL2* significantly correlated to each other (*r* = 0.986, *p <*0.0001). The multivariate analysis demonstrated that serum pentosidine is an independent risk factor for *AGER* expression. With logistic regression analysis, the area under the ROC curve (AUC) for serum pentosidine at the cut-off level of 2.1 ng/ml and FPG at 100 mg/dl, which is a cut-off point for prediabetes, was significantly higher for predicting *AGER* expression than that of serum pentosidine alone (0.803 vs 0.688, *p* = 0.048), indicating that serum pentosidine was a good predictor of *AGER* expression in prediabetic individuals. In conclusion, this study demonstrated a preserved osteogenic differentiation in the PBMC derived from prediabetic individuals. In addition, those PBMC with preserved osteogenic differentiation potential showed the suppression of both cellular RAGE and apoptotic-related signals. Serum pentosidine was an independent risk factor for cellular RAGE expression and is conceivably a good predictor for *AGER* suppression in prediabetic individuals.

## Introduction

Type 2 diabetes is a health problem of great concern worldwide. It is a metabolic disorder that has insulin resistance as a central pathophysiology. That insulin resistance occurs many years before the development of progressive dysglycemia, starting from modest hyperglycemia in prediabetes to full-blown chronic hyperglycemia in type 2 diabetes. According to the American Diabetes Association, prediabetes can be diagnosed by one of the three following criteria (1): first, impaired fasting glucose (IFG) defined by fasting plasma glucose (FPG) of between 100 and 125 mg/dl; second, impaired glucose tolerance (IGT) defined by FPG of between 140 and 199 mg/dl at 2 h after 75 g of oral glucose loading; and third, a level of glycated hemoglobin (HbA1c) that is between 5.7 and 6.4% (1). It is well documented that chronic hyperglycemia accelerated the accumulation of advanced glycation end products (AGEs) in many tissues, influencing the occurrence of chronic microvascular and macrovascular complications found in patients with diabetes (2–5). Even though prediabetes is a condition with hyperglycemia which is lower than the threshold for the diagnosis of diabetes, prediabetes can also lead to similar chronic microvascular and macrovascular complications to those found in individuals with diabetes (6, 7). However, there is no evidence to show the association between the accumulation of AGEs and those complications found in prediabetes. Moreover, Gateva and colleagues (8) demonstrated that serum pentosidine, a type of AGEs, did not relate to vascular complications in prediabetic individuals.

Type 2 diabetic individuals consistently showed an impaired bone quality represented by a decreased bone turnover (9, 10), deteriorated bone microarchitecture (11), and an increased risk of fragility fractures (12–15) even though they had a preserved bone mineral density (13–15). Individuals with prediabetes who have a milder level of hyperglycemia than those with diabetes were also shown for low bone turnover (16, 17) and preserved bone mineral density (17–20). However, evidence involving impairment of bone quality and fragility fractures in prediabetic individuals is still controversial. Chen and colleagues (19) and Park and colleagues (21) demonstrated an increased risk of hip fractures in a prediabetic population. In contrast, Dominic and colleagues (22), and Iki and colleagues (23), demonstrated that hip fractures did not increase in a population with prediabetes. In addition, Dowson-Hughes and colleagues (24) showed the preservation of bone material strength in individuals with prediabetes.

AGEs exert their downstream signaling cascades, including inflammatory signaling pathways and apoptotic pathway, *via* interacting with their specific receptors, the receptor of advanced glycation end product (RAGE) (25). The polymorphism of the RAGE gene is associated with proinflammation and oxidative stress, and also with diabetic retinopathy, a microvascular complication of diabetes (26). The activation of RAGE lead to dysfunction and apoptosis of cells, namely, osteogenic lineage cells (27–32). The accumulation of AGEs conceivably contributes to the impairment of bone quality found in type 2 diabetes. Preclinical studies have shown that the skeletal accumulation of AGEs inhibited osteoblast differentiation (28–30), enhanced osteoblast apoptosis (27, 29–31), and deteriorated the mechanical properties of the skeleton (33–35). In human subjects, our previous studies showed that the peripheral blood mononuclear cells (PBMC) derived from patients with type 2 diabetes had an impaired osteogenic differentiation potential which could be linked to the overexpression of RAGE (36, 37). In addition, serum pentosidine has been shown to have a positive correlation with vertebral fractures in patients with type 2 diabetes (38–40). Although AGEs have been widely documented for their detrimental effects on bone cells and bone strength in type 2 diabetes, the effects of AGEs on the skeleton remain inconclusive in prediabetes. To date, there have only been a few preclinical studies that demonstrated an adverse effect of being prediabetic on osteoblast function and the skeletal microarchitecture. Pramojanee and colleagues (41) showed a decrease in osteoblast proliferation and survival, and a deterioration of bone microarchitecture in prediabetic rats. In addition, Ross and colleagues (42) demonstrated a decreased osteoblast differentiation and compromised cortical bone microarchitecture in prediabetic mice. However, both studies did not explore the effects of AGEs on those cellular dysfunction and skeletal microarchitecture deterioration. To date, there is no evidence to show osteoblast dysfunction and the skeletal impacts of AGEs in patients with prediabetes. Therefore, it remains to be elucidated whether an impairment of osteogenic differentiation or an alteration of cellular RAGE expression occurs in prediabetic patients.

Mesenchymal stem cells can be derived from various adult tissues including adipose tissue, bone marrow and peripheral blood (43). It is well documented that the peripheral blood-derived mesenchymal stem cells (PB-MSC) can differentiate into multiple cell types, namely, adipocytes, chondrocytes, and osteoblasts (44–46). In terms of osteoblast differentiation, Valenti and colleagues (47) demonstrated the expression of multiple osteoblast-specific genes during differentiation of the PB-MSC, namely, *COL1A1* and *RUNX2*, and also the production of the *BGALP*-encoded protein named osteocalcin. In addition, our previous studies also demonstrated the differentiation toward osteoblast of the PBMC-isolated from both non-diabetic and diabetic individuals (36, 37). Therefore, to obtain those stem cells with the least invasive measure, this study was conducted using the PBMC-isolated from participants to investigate the osteogenic differentiation potential of the stem cells. This study aimed to determine whether 1) there was an osteogenic differentiation defect in PBMC derived from prediabetic individuals, and 2) there was an alteration of RAGE expression in PBMC isolated from patients with prediabetes.

## Materials and Methods

### Ethics Statement

This study was a cross-sectional study, performed at the Maharaj Nakorn Chiang Mai Hospital, Chiang Mai University, Chiang Mai, Thailand and approved by the Research Ethics Committee of the Faculty of Medicine, Chiang Mai University. All participants provided their written informed consent to participate in this study before enrollment.

### Study Population and Sample Collection

Individuals with FPG between 100 and 125 mg/dl on at least two occasions, which were classified as impaired fasting glucose (IFG) by the American Diabetes Association, were enrolled as prediabetic subjects. Age-matched individuals with FPG less than 100 mg/dl were enrolled as normoglycemic subjects. Individuals with IFG were excluded if HbA1c higher than 6.4%. The other exclusion criteria were as follows: females with serum creatinine higher than 1.4 mg/dl males with serum creatinine above 1.5 mg/dl; individuals who use steroids, anti-resorptive agents or anabolic agents for osteoporosis, immunosuppressive agents, thiazolidinedione; and individuals with hematologic or metastatic malignancy. Venous blood (35–40 ml) was collected from all enrolled participants to isolate the PBMC, and to determine serum levels of pentosidine (Elabscience Biotechnology, WuHan, Hubei, China), soluble RAGE (sRAGE) (R&D, Minneapolis, MN, USA), interleukin 1-β (IL1-β) (R&D, Minneapolis, MN, USA) and tumor necrosis factor-α (TNF-α) (R&D, Minneapolis, MN, USA) by ELISA. FPG, HbA1c, serum creatinine, high-density lipoprotein cholesterol (HDL-C), low-density lipoprotein cholesterol (LDL-C), and triglyceride levels were assessed using standardized procedures at the central laboratory of the Faculty of Medicine, Chiang Mai University. Glomerular filtration rate (eGFR) was calculated using the Chronic Kidney Disease Epidemiology Collaboration (CKD-EPI) method. Fracture risk estimation was estimated from the Fracture Risk Assessment Tool (FRAX^®^) using the Thailand database (48).

### Human Peripheral Blood-Derived Mononuclear Cells (PBMC) Isolation and Culture Protocol

Peripheral venous blood (35–40 ml) was applied to density gradient centrifugation and PBMC were isolated and then cultured as described in our previous study (36). In brief, the plasma was removed from the venous blood by centrifugation at 1,500 rpm for 5 min. The remaining cell fraction was first diluted with an equal volume of DMEM (Gibco, Grand Islands, NY, USA) and then overlaid on Histopaque (specific gravity 1.077 g/ml; Sigma-Aldrich, St. Louis, MO, USA) and further centrifuged at 4,000 rpm for 30 min. The cells in the mononuclear cell layer (PBMC) were plated in 24-well culture plates and cultured in RPMI supplemented with 10% (v/v) fetal bovine serum (Gibco, Grand Islands, NY, USA). The floating cells in the culture wells were removed and the plastic-adhered cells were further cultured in a non-osteogenic-inducing medium, DMEM supplemented with 10% (v/v) fetal bovine serum (Gibco, Grand Islands, NY, USA), for 7–10 days until confluence. To induce osteogenic differentiation, the adhered cells were cultured in a non-osteogenic-inducing medium until reaching 50% confluence. They were then transferred to an osteogenic-inducing medium; DMEM supplemented with 10^−7^ M dexamethasone, 60 μM ascorbic acid, and 10 mM β-glycerophosphate) and cultured for a further 21 days.

### Analysis of the Expression of Osteoblast-Specific Genes, *AGER*, and Cellular Apoptotic-Associated Genes

The expression of genes, including osteoblast-specific genes, *AGER*, *BAX*, and *BCL2*, was quantified using real-time PCR as described in our previous study (36, 37). In brief, the total RNA (500 ng) was isolated from the cell lysate using the illutraRNA spin Mini Kit (GE Healthcare Life Science, Buckinghamshire/Little Chalfont, UK) following the manufacturer’s instructions. The isolated total RNA of each sample was reverse transcribed into cDNA using an iScript™cDNA Synthesis Kit (Bio-Rad, Hercules, CA, USA) following the manufacturer’s protocol. The cDNA was subsequently analyzed by reversed transcription PCR (RT-PCR) using Sso7d fusion enzyme technology according to the manufacturer’s instruction (Bio-Rad, Hercules, CA, USA). The PCR protocol consisted of 45 cycles of 5 s at 95°C, 10 s at 60°C, and 30 s at 72°C using the Applied Biosystems 7500/7500 Fast Real-Time PCR system. The total RNA isolated from both non-osteogenic and osteogenic-inducing cells was used to analyze: 1) osteoblast-specific genes, namely, *ALPL*, *BGLAP*, *COL1A1*, and *RUNX2* for representing osteoblast differentiation and 2) PPAR-γ which is a transcription factor driving towards adipocytes for evaluating signals against osteoblast differentiation. In this study, since multiple osteoblast-specific genes, including *ALPL*, *BGLAP*, *COL1A1*, and *RUNX2*, were expressed during the differentiation process toward osteoblasts (47, 49) and were persistently elevated in human osteoblasts (49, 50), the differentiation towards osteoblasts was defined by the increment of expression of all osteoblast-specific marker genes, including *ALPL*, *BGLAP*, and *COL1A1*, and also the increment of the *RUNX2/PPARγ* ratio. In contrast, the total RNA extracted only from the non-osteogenic-inducing cells was used to determine: 1) *AGER* expression to elucidate the alteration of cellular RAGE expression and 2) *BAX* and *BCL2* expression for evaluation of cellular apoptotic signals. The *GAPDH* expression was used for normalization of the relative expression levels for each primer set (**Table 1**) by the 2^(−ΔΔCT)^ method. All primers were purchased from Invitrogen.

**Table 1:** Sequences of Real-Time qPCR Primers.

Genes	Primer sequence (5’-3’)	
	Forward	Reverse
**Osteoblast-specific genes**		
*ALPL*	CATGGCTTTGGGCAGAAGGA	CTAGCCCCAAAAAGAGTTGCAA
*BGLAP*	GAAGCCCAGCGGTGCA	CACTACCTCGCTGCCCTCC
*COL1A1*	CAGCCGCTTCACCTACAGC	TTTTGTATTCAATCACTGTCTTGCC
*RUNX2*	TCTTAGAACAAATTCTGCCCTTT	TGCTTTGGTCTTGAAATCACA
**Adipocyte-specific gene**		
*PPARγ*	AAAGAAGCCAACACTAAACC	CTTCCATTACGGAGAGATCC
**Apoptotic signal-associated genes**		
*BAX*	TGGAGCTGCAGAGGATGATTG	GAAGTTGCCGTCAGAAAACATG
*BCL2*	CATGCTGGGGCCGTACAG	GAA CCGGCACCTGCACAC
**Others**		
*AGER*	GCTGGAATGGAAACTGAACACAGG	TTCCCAGGAATCTGGTAGACACG
*GAPDH*	CCCTTCATTGACCTCAACTA	AGATGATGACCCTTTTGGCT

### Statistical Analysis

Statistical analysis was performed using SPSS version 23.0.All continuous data are reported as mean ± standard deviation while all categorical variables are presented as percentages. An independent *t*-test was used for univariable comparative statistics for continuous data. A Chi-square test was used for univariable comparative statistics for all categorical variables, the exception being the categorical variable with small counts which was analyzed using Fisher’s Exact test. Pearson’s correlation was performed to identify the correlation between two continuous variables. Multivariate linear regression analysis was performed to identify the independent risk factors of *AGER* expression. A logistic regression model was performed to determine the potential for using serum pentosidine as a predictor of *AGER* suppression in prediabetic individuals. The areas under the receiver operating characteristics (ROC) curves of the model (AUC) were plotted to determine the diagnostic performance of the serum pentosidine cut-off value which was calculated based on the highest sensitivity or specificity. A *p*-value of less than 0.05 was used as a measure of statistical significance. A sample size calculation was performed to estimate the number needed to show the non-inferiority of osteogenic differentiation in the prediabetic group compared to the normoglycemic group (51). A sample size of at least 12–16 participants in the normoglycemic group and 17–23 patients in the prediabetic group was estimated to give 80% power at the 5% significance level to detect a non-inferiority of osteogenic differentiation in the prediabetic group compared to the normoglycemic group at a margin of equivalence of 20–25% (36, 51).

## Results

### Demographic Data, Clinical Characters, and Biochemical Parameters of Study Participants

This study included 28 individuals with prediabetes and 17 age-matched participants with normoglycemia. All participants with prediabetes were diagnosed with impaired fasting glucose (IFG) using the FPG criteria with a cut-off value of 100–125 mg/dl as recommended by the American Diabetes Association (1). All prediabetic participants had at least two FPG instances between 100 and 125 mg/dl during the three months before or after the recruitment date. All participants in the normoglycemic group had never had a recorded FPG higher than 99 mg/dl before enrollment. Age, gender, body mass index (BMI), systolic blood pressure (SBP), diastolic blood pressure (DBP), eGFR, HDL-C, LDL-C, triglyceride and 10-year fracture risk as determined by FRAX^®^ using the Thailand database were comparable in both normoglycemic and prediabetic groups (**Table 2**). Either angiotensin-converting enzyme inhibitors (ACEI) or angiotensin II receptor blockers (ARB) were used at a higher rate in patients with prediabetes while dihydropyridine calcium channel blocker (DHP-CCB) was used at a higher rate in participants with normoglycemia; however, the difference did not reach statistical significance (**Table 2**). Lipid-lowering agents, namely, statins and fibrates were used at a comparable rate in both normoglycemic and prediabetic groups (**Table 2**).

**Table 2:** Clinical Characteristics of the Study Participants.

Parameter	Normoglycemia (n = 17)	Prediabetes (n = 28)	*p*-value
Age (years)	59.1 ± 9.0	61.1 ± 7.7	0.424
Gender (% female)	64.7	71.4	0.637
BMI (kg/m²)	24.3 ± 2.6	24.5 ± 3.9	0.876
SBP (mmHg)	126.5 ± 14.5	132.9 ± 12.2	0.124
DBP (mmHg)	80.4 ± 9.4	76.9 ± 10.3	0.261
FPG (mg/dl)	90.2 ± 6.3	102.1 ± 10.7	<0.0001
HbA1c (%)	–	5.9 ± 0.5	–
Triglyceride (mg/dl)	95.6 ± 61.2	119.1 ± 94.2	0.367
LDL-C* (mg/dl)	112.5 ± 42.9	100.9 ± 28.6	0.282
HDL-C# (mg/dl)	62.7 ± 31.4	60.5 ± 14.7	0.751
IFG duration (years)	–	3.4 ± 2.8	–
Drugs (% use)			
* ACEI or ARB**	23.5	48.1	0.102
* DHP-CCB##	58.8	37.0	0.158
* Thiazide-like diuretic	11.8	25.9	0.257
* Statins	52.9	57.1	0.783
* Fibrate	11.8	10.7	0.913
eGFR (ml/min)	76.4 ± 26.7	84.7 ± 21.9	0.264
FRAX: 10-year risk of hip fractures (%) (FRAX-H)	0.7 ± 1.0	1.5 ± 2.1	0.215
FRAX:10-year risk of osteoporotic fractures (%) (FRAX-O)	2.9 ± 1.6	4.6 ± 3.5	0.085

*LDL-C, low-density lipoprotein cholesterol.

^#^HDL-C, high-density lipoprotein cholesterol.

**ACEI angiotensin-converting enzyme inhibitors.

**ARB, angiotensin II receptor blockers.

^##^DHP-CCB, dihydropyridine calcium channel blockers.

In the group with prediabetes, the duration of being prediabetic was 3.4 ± 2.8 years. Patients within the prediabetic group had a significantly higher level of FPG than those in the normoglycemic group (102.1 ± 10.7 mg/dl vs 90.2 ± 6.3 mg/dl, *p <*0.0001) and had a level of hemoglobin A1c (HbA1c) of 5.9 ± 0.5%. All prediabetic patients were recommended for lifestyle modification as a measure for diabetic prevention except one patient received metformin at the dosage of 1,000 mg daily.

Serum pentosidine was slightly lower in the prediabetic group than that in the normoglycemic group (3.0 ± 1.8 ng/ml vs 4.1 ± 2.2 ng/ml, *p* = 0.071); however, the difference did not reach statistical significance (**Table 3**). The level of serum pentosidine positively correlated with the level of HbA1c by univariate analysis (*r* = 0.614, *p* = 0.044) in prediabetic population, suggesting that hyperglycemia had an impact on the accumulation of AGEs. Serum sRAGE, a decoy receptor of AGEs, and also the ratio between sRAGE and pentosidine (sRAGE–pentosidine ratio), were comparable in both prediabetic and normoglycemic groups (**Table 3**). In addition, serum interleukin-1β (IL-1β) and tumor necrosis factor-α (TNF-α), markers of systemic inflammation, were comparable in both prediabetic and normoglycemic groups (**Table 3**). Therefore, individuals in both prediabetic and normoglycemic groups had comparable baseline characteristics (**Tables 2**, **3**); FPG was the only parameter that showed significant difference between the two groups (**Table 2**).

**Table 3:** Serum Pentosidine, sRAGE and Inflammation Markers in the Study Participants.

Parameter	Normoglycemia (n = 17)	Prediabetes (n = 28)	*p*-value
Pentosidine (ng/ml)	4.1 ± 2.2	3.0 ± 1.8	0.071
Soluble RAGE (sRAGE) (pg/ml)	610.4 ± 352.3	539.9 ± 380.7	0.539
sRAGE-Pentosidine ratio (pg/ng)	196.6 ± 181.8	245.6 ± 232.4	0.462
Interleukin-1β (pg/ml)	0.7 ± 0.9	0.7 ± 0.7	0.785
Tumor necrosis factor-α (pg/ml)	1.7 ± 3.6	2.6 ± 2.8	0.319

### Preserved Osteogenic Differentiation, and Suppressed Expression of Both *AGER* and Apoptotic-Related Signals in PBMC Derived From Patients With Prediabetes

**Figure 1:**
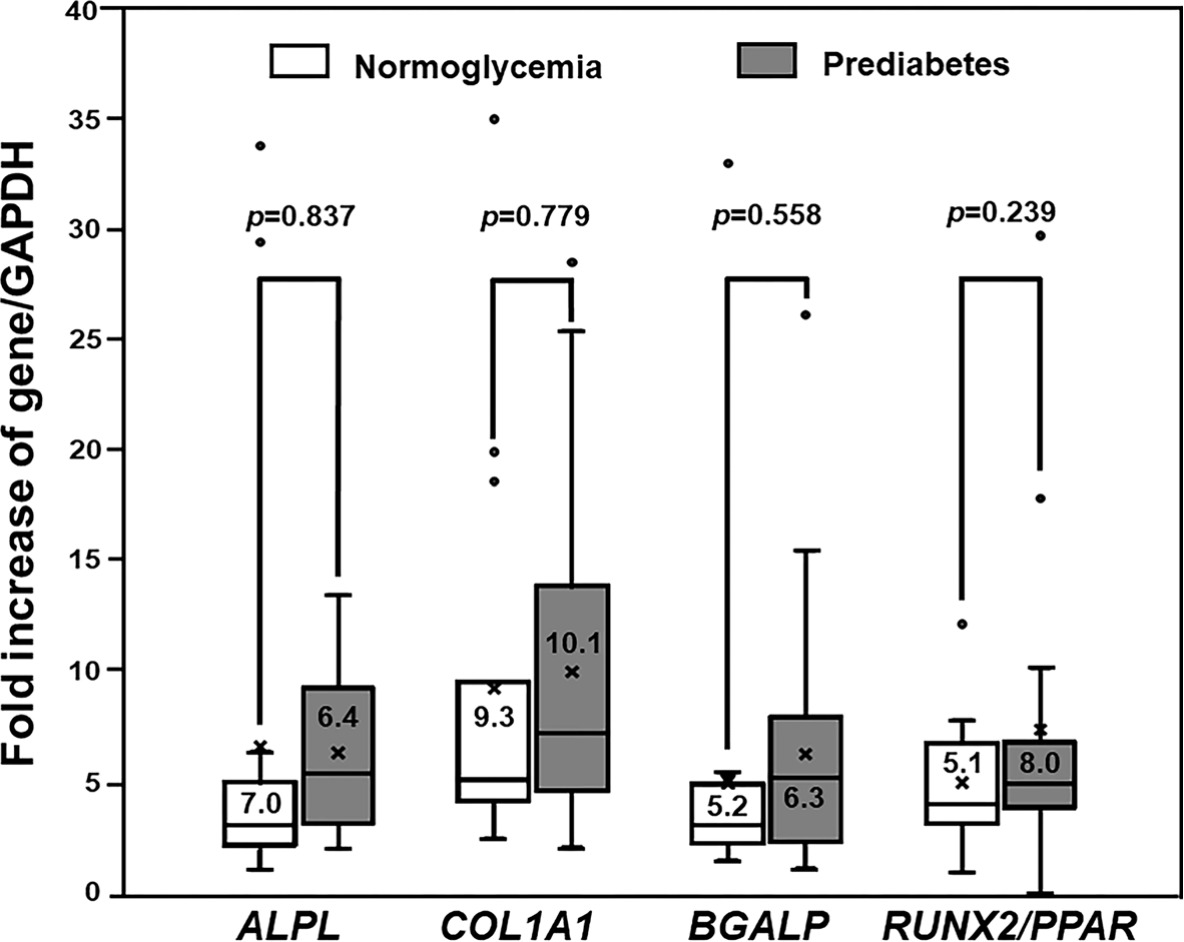
The Expression of Osteogenic Differentiation Markers. Box and whisker plots to show a comparison of the expression of osteoblast-specific genes between participants with normoglycemia and patients with prediabetes (mean ± SD). Both prediabetic and normoglycemic groups had comparable levels of osteoblast-specific gene expression, namely, *ALPL*, *COL1A1*, *BGALP*, and *RUNX2/PPARγ* ratio (*RUNX2/PPAR*).

The levels of expression of multiple osteoblast-specific genes, namely, *ALPL*, *BGLAP*, *COL1A1*, and *RUNX2/PPAR* were measured in the PBMC-isolated from both prediabetic and normoglycemic groups to define osteogenic differentiation. The PBMC derived from patients with prediabetes showed a preservation of osteogenic differentiation potential. The PBMC-isolated from 25 patients with prediabetes (25/28) expressed *ALPL*, *BGLAP*, *COL1A1*, and *RUNX2/PPAR* and the PBMC-isolated from 15 patients with normoglycemia (15/17) expressed those genes. Therefore, the PBMC derived from both prediabetic and normoglycemic groups had a comparable rate of osteogenic differentiation (89.3 and 88.2%, *p* = 1.000). Among the PBMC showing differentiation toward osteoblast, the levels of osteoblast-specific gene expression were not significantly different between the normoglycemic and prediabetic groups, namely, *ALPL* (7.0 ± 10.2 vs 6.4 ± 3.5, *p* = 0.837), *COL1A1* (9.30 ± 9.2 vs 10.1 ± 7.3, *p* = 0.779), *BGALP* (5.2 ± 7.8 vs 6.3 ± 5.5, *p* = 0.588), and *RUNX2/PPAR* (5.1 ± 3.0 vs 8.0 ± 7.6, *p* = 0.239) (**Figure 1**). According to the results of osteogenic differentiation in this study, we further performed backward power calculations to confirm that our sample size had adequate power to demonstrate a comparable differentiation ability between the prediabetic and normoglycemic groups (51). As a result, our study had 80% power to demonstrate non-inferior potential for osteogenic differentiation in the prediabetic group compared to the normoglycemic group at a margin of equivalence of 23%.

**Figure 2:**
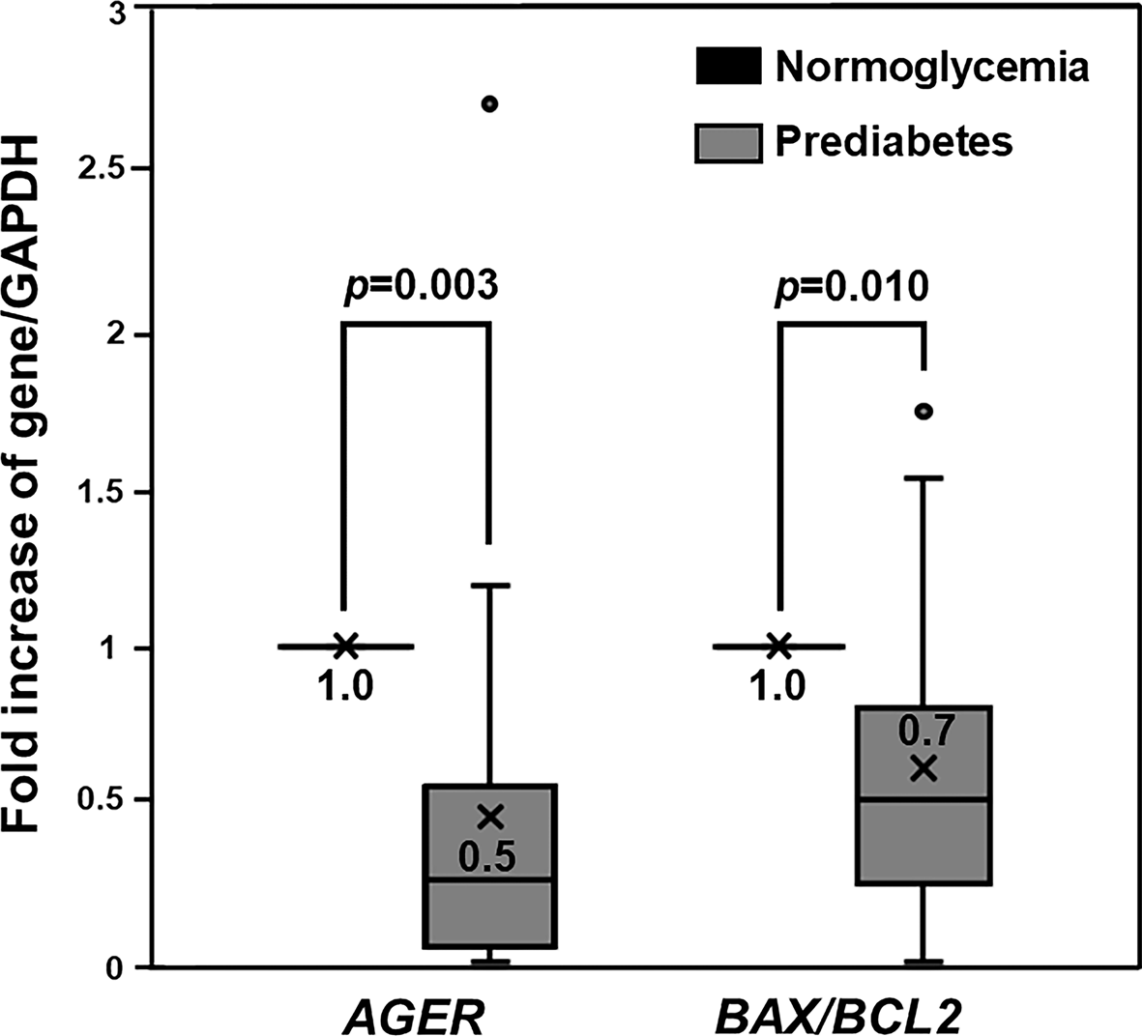
The Expression of *AGER*, *BAX*, and *BCL2* Genes. Box and whisker plots to show a comparison of *AGER* and *BAX/BCL2* ratio between PBMC-isolated from individuals with normoglycemia and prediabetes. *AGER* and *BAX/BCL2* ratio in PBMC-isolated from individuals with prediabetes were significantly suppressed by 2 and 1.4 folds, respectively, compared to those in age- and pentosidine-matched PBMC-isolated from participants with normoglycemia.

Our previous studies demonstrated impaired osteogenic differentiation in PBMC isolated from patients with type 2 diabetes (36, 37), as well as cellular RAGE overexpression only in PBMC showing an impaired ability to differentiate toward osteoblast (37). Therefore, that cellular RAGE overexpression was conceivably associated with the impairment of osteogenic differentiation found in patients with type 2 diabetes. Since we demonstrated a preservation of osteogenic differentiation potential in PBMC derived from prediabetic patients, it was interesting to determine how cellular RAGE expression changes in those cells. To determine the alteration of cellular RAGE expression in patients with prediabetes showing preserved osteogenic differentiation, the level of *AGER* gene expression was compared between groups with prediabetes and normoglycemia. The PBMC showing poor osteogenic differentiation, two in the normoglycemic group and three in the prediabetic group, were excluded from the following analysis due to very small counts in these groups. Since pentosidine has been documented in other studies as an *AGER* enhancer (28, 29), and age was demonstrated in our previous study as a predictor of osteogenic differentiation potential (37), the level of *AGER* expression in prediabetic participants was compared with the level of *AGER* expression in age- and pentosidine-matched normoglycemic individuals. Interestingly, the *AGER* expression was significantly suppressed in individuals with prediabetes compared to age- and pentosidine-matched normoglycemic individuals (0.5 ± 0.6 vs 1.0 ± 0.0, *p* = 0.003) (**Figure 2**). Since the activation of RAGE gives rise to the activation of its downstream apoptotic pathway, the levels of *BAX* and *BCL2* expression were measured to determine whether the apoptotic-associated signals were also suppressed in prediabetic individuals. In concordant with *AGER* expression, *BAX/BCL2* expression ratio was significantly suppressed in prediabetic participants compared to those with normoglycemia (0.7 ± 0.5 vs 1.0 ± 0.0, *p* = 0.010) (**Figure 2**). Furthermore, the level of *AGER* expression significantly correlated with the level of the *BAX/BCL2* expression ratio (*r* = 0.986, *p <*0.0001). Therefore, the cellular RAGE suppression shown in prediabetes was conceivably a protective factor against cellular apoptosis and also osteogenic differentiation defects.

### Serum Pentosidine was an Independent Risk Factor for Determining *AGER* Expression in Individuals With Prediabetes

Since the lower level of *AGER* expression was conceivably associated with the preservation of osteogenic differentiation of PBMC isolated from prediabetic individuals, the factors influencing that *AGER* suppression were valuable to be elucidated. Univariate analysis showed that *AGER* expression positively correlated with serum pentosidine (*r* = 0.513, *p* = 0.006), but inversely correlated with the duration of being prediabetic (*r* = −0.480, *p* = 0.011). Next, we performed the multivariate analysis using backward linear regression analysis to identify an independent risk factor for the expression of *AGER*. In addition to serum pentosidine and duration of being prediabetic, we included another 4 parameters that showed a correlation with either serum pentosidine or duration of being prediabetic in the further analysis because those factors may influence the expression of *AGER*. These 4 factors were as follows: HbA1c, which showed a positive correlation with serum pentosidine in the prediabetic group (*r* = 0.614, *p* = 0.044); and FPG (*r* = 0.425, *p* = 0.007), FRAX-H (*r* = 0.477, *p* = 0.004), and FRAX-O (*r* = 0.49, *p* = 0.003), which showed a positive correlation with the duration of being prediabetic. Upon performing the backward linear regression analysis, the multivariate analysis showed that only serum pentosidine significantly associated with the expression of *AGER* (*r* = 0.879, *p* = 0.002), indicating that serum pentosidine was an independent risk factor for determining *AGER* expression. Therefore, in this present study, serum pentosidine was the only factor identified as independently contributing to the expression of *AGER.*

**Figure 3:**
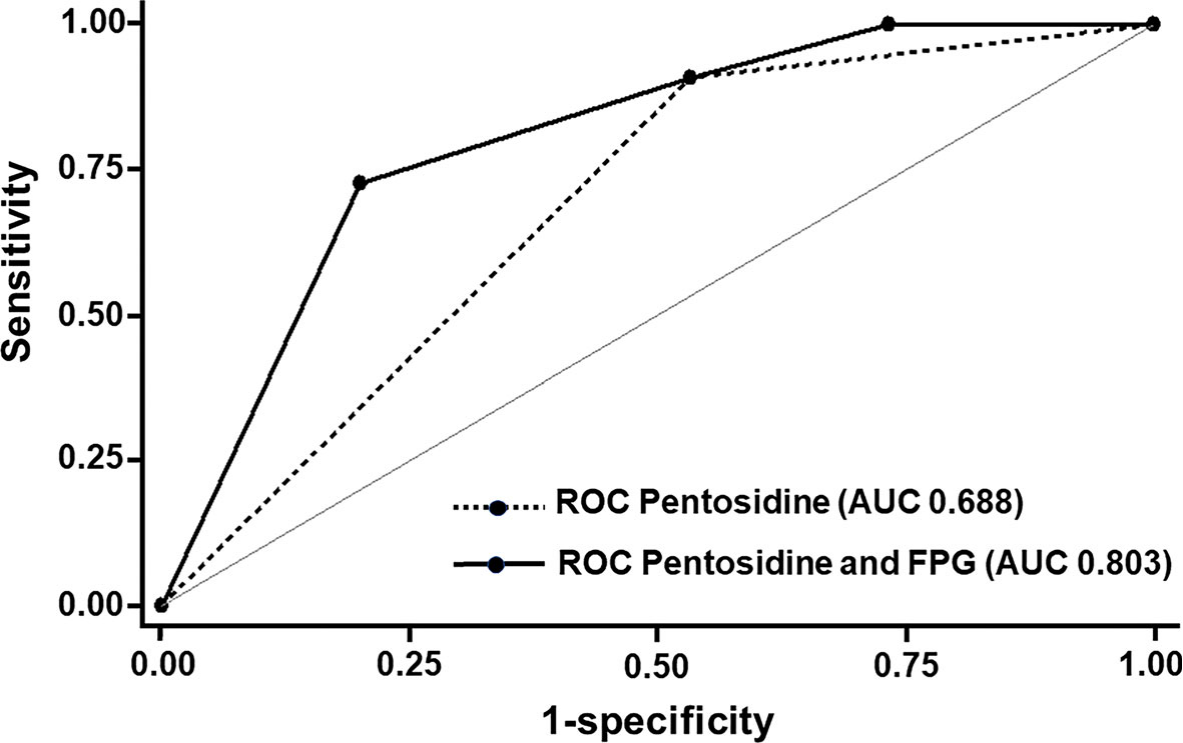
ROC and Area under ROC (AUC) for the Prediction of the expression of *AGER.* With **s**erum pentosidine at the cut-off level of 2.1 ng/ml, the AUC of serum pentosidine to predict the expression of *AGER* was 0.688. With the combination of both serum pentosidine at the cut-off level of 2.1 ng/ml and fasting plasma glucose at the cut-off level of 100 mg/dl into the model, the AUC significantly increased from 0.688 to 0.803 (0.803 vs 0.688, *p* = 0.048).

We next determined the potential of using serum pentosidine as a predictor of *AGER* suppression in prediabetic individuals. We categorized the *AGER* expression into two groups, including the *AGER* suppression group as defined by an *AGER* expression level of less than 1 and the *AGER* non-suppression group as defined by the level of *AGER* expression as equal to 1 or higher. Regarding serum pentosidine, the cut-off level of serum pentosidine to predict the level of *AGER* expression with the highest sensitivity and specificity was calculated. As a result, the cut-off level of serum pentosidine to predict the level of *AGER* expression with 90.9% sensitivity and 46.7% specificity was 2.1 ng/ml. Therefore, we applied this value of serum pentosidine for further analysis. After categorizing patients into two groups according to the level of *AGER* expression, we next performed logistic regression analysis to determine whether the proposed cut-off point of serum pentosidine predicted the occurrence of *AGER* suppression. When the *AGER* expression was categorized into two groups, serum pentosidine at the cut-off level of 2.1 ng/ml (OR 8.75; 95% CI 0.88–86.6, *p* = 0.064) and FPG at the cut-off level of 100 mg/dl (OR 0.15; 95% CI 0.02–0.94, *p* = 0.043) showed an association with the group of *AGER* expression. As demonstrated in **Figure 3**, the area under the ROC curve (AUC) of serum pentosidine to predict the *AGER* expression was 0.688. In addition to serum pentosidine, when we put the cut-off point of FPG at the prediabetic range into the model, the AUC significantly increased from 0.688 to 0.803 (0.803 vs 0.688, *p* = 0.048) (**Figure 3**). This improvement in AUC indicated that serum pentosidine was a good predictor for *AGER* expression in prediabetic patients. The predictive probability of *AGER* suppression by serum pentosidine at the cut-off point of less than 2.1 ng/ml and FPG at the cut-off point of prediabetes was 0.97, which means that 97% of patients with prediabetes were correctly classified into the *AGER* suppression group if those patients had a serum pentosidine level lower than 2.1 ng/ml.

The results of linear regression analysis indicated that serum pentosidine was an independent risk factor for *AGER* expression. However, from the result of logistic regression analysis, serum pentosidine was shown as a good predictor for *AGER* expression only in the prediabetic group. Therefore, our study demonstrated the potential of using serum pentosidine as a predictor of *AGER* expression in prediabetic individuals. With a serum pentosidine level of lower than 2.1 ng/ml, there was an extremely high possibility of the expression of *AGER* being suppressed in prediabetic individuals. Since the *AGER* suppression shown in the prediabetic group was conceivably a protective factor against cellular apoptosis as well as osteogenic differentiation defects, it may be inferred that a serum pentosidine level of lower than 2.1 ng/ml is a protective factor against cellular apoptosis and impaired osteogenic differentiation in this prediabetic group.

## Discussion

This study demonstrated the preservation of osteogenic differentiation ability in the PBMC derived from prediabetic individuals as defined by the impaired fasting glucose criteria. In addition, the suppression of *AGER* and apoptotic-related signals was demonstrated in those prediabetic-derived PBMC that showed the preservation of osteogenic differentiation potential. Serum pentosidine was shown as an independent risk factor for the cellular RAGE expression. In addition, the level of serum pentosidine of lower than 2.1 mg/dl was demonstrated as being a good predictor for the occurrence of *AGER* suppression in individuals with prediabetes.

In high fat diet-induced obesity and prediabetic animal models, a decrease in osteoblast proliferation, differentiation, and survival, and also a deterioration of bone microarchitecture have been consistently demonstrated (41, 42). However, in humans, it has been shown that individuals with prediabetes retain a preservation of both bone mineral density (17–20) and bone material strength (24). Moreover, there have been inconsistent reports involving the increment of hip fracture in prediabetic population (19, 21–23). Therefore, the detrimental effects of prediabetes on the skeleton remain to be elucidated in prediabetic patients. This study involving osteogenic differentiation ability of the stem cells in prediabetic patients may give rise to additional information on this obscure issue. Furthermore, it is valuable to elucidate whether there is an osteogenic differentiation defect at the prediabetic stage as a measure for establishing a skeletal health strategy in type 2 diabetes in the future. In this study, we demonstrated the preservation of osteogenic differentiation in PBMC isolated from patients with prediabetes. The PBMC derived from this prediabetic group showed the same rate of differentiation toward osteoblasts as the rate in the normoglycemic group. In addition, they also had the same degree of osteoblast-specific gene expression as that in normoglycemic individuals. Therefore, being prediabetic as diagnosed by IFG, did not show a detrimental effect on the osteogenic differentiation potential of the PBMC, which is probably linked to the preservation of bone mineral density and bone microarchitecture which has been shown in several previous studies and may favor a neutral fracture risk in prediabetic individuals.

Our previous study, performed in patients with type 2 diabetes, demonstrated *AGER* overexpression only in PBMC showing poor osteogenic differentiation ability but not in the PBMC showing preserved osteogenic differentiation (37), suggesting a linkage between *AGER* overexpression and a defect in osteogenic differentiation in PBMC derived from individuals with type 2 diabetes. In this study, we demonstrated the preservation of osteogenic differentiation in the PBMC isolated from patients with prediabetes. Therefore, it is interesting to see how cellular RAGE expression alteration in this group of patients with preserved osteogenic differentiation. In this study, when we performed analysis of cellular RAGE expression in PBMC showing preserved osteogenic differentiation, we demonstrated a lower level of *AGER* expression in the PBMC derived from the prediabetic group compared to those from the normoglycemic group, indicating cellular RAGE suppression in prediabetes with preserved osteogenic differentiation ability. Therefore, it is noteworthy to state that the level of cellular RAGE expression would influence osteogenic differentiation in both prediabetic and diabetic individuals, ranging from suppression in prediabetes to overexpression in type 2 diabetes. Our results regarding *AGER* suppression in prediabetes were consistent with a previous report (52). Ruelas and colleagues (52) demonstrated lower expression levels of both the *AGER* gene and RAGE protein in PBMC derived from patients with prediabetes and insulin resistance than those in the PBMC-isolated from normal healthy volunteers. However, they did not determine the differentiation potential of those isolated PBMC (52). Even though *AGER* gene suppression was repetitively shown in both our and previous studies, the underlying mechanism leading to the suppression of *AGER* in prediabetes remains to be elucidated. To date, several signal alterations have been shown to maintain normal bone metabolism in prediabetes. In an animal model, Mohammad and colleagues (53) reported a ligand-induced downregulation of TLR4 in bone marrow-derived macrophages isolated from prediabetic mice while TLR4 was constantly high in diabetic mice. Zhang and colleagues (54) demonstrated that the decrement of TLR4 reduced hyperglycemia-induced osteoblast apoptosis, promoted bone mineralization, and improved bone structure in a rat model. Therefore, the downregulation of TLR4 occurred at a prediabetic stage to maintain osteoblast survival and bone integrity. In contrast, Bhansali and colleagues (55) demonstrated an increase in mitophagy-related markers and mitochondrial mass in PBMC derived from patients with prediabetes but showed a decrease in those mitophagy-related markers and mitochondrial mass in cases with type 2 diabetes. Later, Gao and colleagues (56) showed that the regulation of mitochondrial stress by the prevention of oxidative damage to the mitochondria is required during osteogenic differentiation and bone formation. In addition, Dobson and colleagues (57) demonstrated that mitochondrial dysfunction entailed impaired osteogenesis and accelerated age-related osteoporosis. Therefore, the upregulation of mitophagy which occurred at the prediabetic stage was essential to maintain normal osteoblast development and function. Following previous reports on changes in signal regulation in prediabetes, we hypothesized that the downregulation of AGEs-dependent RAGE activation occurred at the prediabetic stage. The downregulation of AGEs-dependent RAGE activation would yield a lower degree of RAGE stimulation even in the presence of the same amount of AGEs, as demonstrated by the significant suppression of *AGER* in prediabetic individuals compared to age- and pentosidine-matched normoglycemic participants in our study. In addition, the downregulation of AGEs-dependent RAGE activation may lead to a lower rate of AGEs formation even in the presence of mild hyperglycemia, as demonstrated by the insignificant lower serum pentosidine level in the prediabetic group compared to the normoglycemic group in our study. Even though the underlying mechanism for the downregulation of AGEs-dependent RAGE activation remains to be further elucidated, it is noteworthy to state that the downregulation of AGEs-dependent RAGE activation that occurred at the prediabetic stage is conceivably one of the mechanisms to promote osteoblast differentiation and survival, which then maintains osteogenesis and bone integrity in individuals with prediabetes.

Since the suppression of *AGER* conceivably contributes to a decrease in cellular apoptosis and preservation of osteogenic differentiation in prediabetes, the suppression of *AGER* would be a good predictive factor for the maintenance of skeletal integrity in prediabetes. However, the measurement of *AGER* expression in the PBMC required multiple steps that were mostly performed on research scales. Further analysis was conducted to determine the independent risk factors of *AGER* expression. If this independent risk factor could be analyzed easily, it may be used as a surrogate marker for cellular RAGE expression and the differentiation ability of the PBMC. Using linear regression analysis, serum pentosidine was shown to be an independent risk factor for *AGER* expression. Since the cut-off point of serum pentosidine level to predict the adverse outcomes of AGEs has never been reported, the cut-off point of serum pentosidine was calculated for the highest sensitivity and specificity in our study. A serum pentosidine level of 2.1 ng/ml showed the highest sensitivity and specificity to predict the expression of *AGER*. Logistic regression analysis was then performed to determine the predictive potential of this proposed cut-off point. Our results showed that this proposed cut-off point was a good predictor for *AGER* expression only in the prediabetic group (AUC 0.803). Since serum pentosidine is much easier to measure than cellular RAGE expression, it is more applicable, especially in the clinic, to use serum pentosidine as a surrogate marker for cellular RAGE expression and differentiation potential of the PBMC. Serum pentosidine has previously been shown to be useful as a predictor of chronic microvascular (2, 4) and macrovascular complications (3), and also vertebral fractures (14, 39, 40) in a diabetic population. A higher level of serum pentosidine would increase the risk of diabetic complications and fracture; however, a cut-off point for clinical prediction has never been proposed. Our study provided another aspect of using serum pentosidine as a protective predictive factor in prediabetes. A level of serum pentosidine lower than the threshold of 2.1 ng/ml was found to be associated with lower *AGER* expression level and may relate to the preservation of osteogenic differentiation in prediabetes.

This study is the first study to demonstrate the preservation of osteogenic differentiation in PBMC derived from prediabetic individuals, and being the first to show the suppression of cellular RAGE and apoptotic-related signals in those PBMC with a preserved osteogenic differentiation ability. In addition, this study proposes the possibility of using serum pentosidine as a surrogate marker for cellular RAGE expression and osteogenic differentiation ability in prediabetic patients. However, this evidence should be interpreted with caution as there are several limitations. First, this study only demonstrated signal activation by mRNA levels due to the limited number of isolated cells from the relatively small 35–40 ml sample of peripheral blood collected from recruited patients. Due to the possibility of the gene being transcripted but not translated into proteins, the *AGER* suppression probably does not lead to lower RAGE activation. Second, this study was a small study, including 28 individuals with prediabetes and 17 participants with normoglycemia, which may have impacted the power of the study. This study was shown to have 80% power to demonstrate non-inferior potential for osteogenic differentiation in the prediabetic group compared to the normoglycemic group at a margin of equivalence of 23%, so the small difference in osteogenic differentiation potential of less than 23% would not be detected by this study. Third, this study demonstrated only the pattern of association between parameters, so the causes and effects of those parameters cannot be concluded. Regarding the association between *AGER* suppression and osteoblast differentiation, studies involving RAGE overexpression would clarify whether cellular RAGE suppression directly yields to preserved osteoblast differentiation. Fourth, the cut-off level of serum pentosidine may not be universally equal to 2.1 ng/dl. This proposed threshold may need to be verified before diagnostic application in the clinic. Even though the ELISA test kit used for the measurement of serum pentosidine in this study has a wide range of detection (0.47–50 ng/dl) and has a coefficient of variation of less than 10% according to the manufacturer information, this test kit is established for a research scale but not for diagnostic purposes. Last, this study was a cross-sectional study which had several unexpected confounding factors caused by the nature of this type of study. Even though all baseline characteristics of the enrolled participants were generally comparable, those unexpected confounding factors might influence the results of the study.

## Data Availability Statement

The original contributions presented in the study are included in the article/supplementary material. Further inquiries can be directed to the corresponding author.

## Ethics Statement

The studies involving human participants were reviewed and approved by the Research Ethics Committee of the Faculty of Medicine, Chiang Mai University. The patients/participants provided their written informed consent to participate in this study.

## Author Contributions

MP was involved in conceptualization, funding acquisition, methodology, formal analysis, original draft writing, reviewing and editing of the manuscript. PP was involved in the methodology, original draft writing, reviewing and editing of the manuscript. NC was involved in funding acquisition, and reviewing and editing of the manuscript. PK was involved in the reviewing and editing of the manuscript. All authors listed have made a substantial, direct, and intellectual contribution to the work and approved it for publication.

## Funding

This work is supported by the Thailand Research Fund MRG5480270 (MP), Merck (MP), the NSTDA Research Chair Grant from the National Science and Technology Development Agency (NC), and the Chiang Mai University Center of Excellence Award (NC). All funders had no role in study design, data collection and analysis, preparation of the manuscript, or decision to publish.

## Conflict of Interest

The authors declare that the research was conducted in the absence of any commercial or financial relationships that could be construed as a potential conflict of interest.

## Publisher’s Note

All claims expressed in this article are solely those of the authors and do not necessarily represent those of their affiliated organizations, or those of the publisher, the editors and the reviewers. Any product that may be evaluated in this article, or claim that may be made by its manufacturer, is not guaranteed or endorsed by the publisher.
